# *In Silico* screening of circulating tumor DNA, circulating microRNAs, and long non-coding RNAs as diagnostic molecular biomarkers in ovarian cancer: A comprehensive meta-analysis

**DOI:** 10.1371/journal.pone.0250717

**Published:** 2021-04-26

**Authors:** Linlin Zhang, Chenyan Hu, Zhongping Huang, Zhijia Li, Qin Zhang, Yang He

**Affiliations:** 1 Department of Gynecology, People’s Hospital of Mianzhu City, Deyang, Sichuan, China; 2 College of Medical Technology, State Key Laboratory of Southwestern Chinese Medicine Resources, Chengdu University of Traditional Chinese Medicine, Chengdu, Sichuan, China; Indiana University School of Medicine, UNITED STATES

## Abstract

**Background:**

Ovarian cancer (OC) is a leading cause of death in gynecological malignancies worldwide. Multitudinous studies have suggested the potential of circulating tumor DNA (ctDNA), circulating microRNAs (miRNAs), and long non-coding RNAs (lncRNAs) as novel diagnostic molecular biomarkers for OC. Here, we include three updated meta-analysis methods using different molecular biomarkers to evaluate their discriminative value in OC diagnosis.

**Methods:**

We conducted three meta-analyses after searching different databases, and 23 eligible articles, including 8 concerning ctDNA, 11 concerning miRNAs, and 4 concerning lncRNAs, were found. Further, we pooled data concerning the sensitivity, specificity, and other indicators of accuracy for ctDNA/miRNAs/lncRNAs in the diagnosis of OC. The heterogeneity was further explored by meta-regressions and subgroup analyses, and Deeks’ funnel plots were used to measure the publication bias of these three meta-analyses.

**Results:**

In all, this meta-analysis included 1732 OC patients and 3958 controls. The sensitivity of ctDNA for OC diagnosis was superior to that of lncRNA and miRNA (84% vs. 81% vs. 78%). Moreover, the specificity and area under the receiver-operating characteristic (ROC) curve (AUC) of ctDNA were 91% and 94%, which were significantly higher than those of miRNA and lncRNAs (78% and 85%; 78% and 86%, respectively). No significant difference was observed among the two meta-analyses of ctDNA and lncRNA (P > 0.05) with regard to publication bias, while the meta-analysis of miRNA observed a significantly small publication bias (P < 0.05).

**Conclusion:**

ctDNA/miRNAs/lncRNAs may be promising molecular biomarkers for OC diagnosis. Further large-scale studies are needed to verify the potential applicability of ctDNA/miRNAs/lncRNAs molecular signatures alone or in combination as diagnostic molecular biomarkers for OC.

## Introduction

Ovarian cancer (OC), one of the three major gynecological malignancies, is a leading cause of death among gynecological malignancies worldwide and accounts for 4% of all cancers in females [1, 2]. Because the ovary is deep within the pelvis, the disease is insidious and asymptomatic at early stages (Stage I or II). Approximately 70% of cases of OC are found to be advanced (Stage III or IV), and the 5-year survival rate for patients is less than 30% [3, 4]; however, this percentage can rise sharply to 92.7% at early stages of OC [5]. Thus, the earlier the precise diagnosis, the more effectively the disease can be controlled at early stages. Therefore, sensitive and specific diagnostic methods or molecular signatures for the early detection of OC are urgently needed to improve overall patient survival.

Traditionally, the gold standard for OC diagnosis with accurate test results is histopathological examination. However, histopathological analysis is not suitable for the early diagnosis of OC because of it is invasive in nature and time consuming [6]. Currently, serum carbohydrate antigen 125 (CA125), the most common serum marker in OC, is being used to assist in the diagnosis of advanced OC and to monitor OC recurrence. However, only 50% of early-stage patients have elevated serum CA125 levels; furthermore, 1% of healthy females, 3% of females with benign ovarian tumors, and 6% females with non-ovarian benign diseases may also report elevated serum CA125 levels. Thus, the sensitivity and specificity of serum CA125 in the early diagnosis of OC are limited and the false-positive rate of serum CA125 is high [7], which limits its application in the early diagnosis of OC. Accordingly, sensitive and specific noninvasive diagnostic molecular biomarkers OC diagnosis are urgently required to improve the prognosis of patients with OC.

Circulating tumor DNA (ctDNA), a part of cell-free DNA (cfDNA), originates from DNA fragments produced by apoptosis, necrosis, or secretion of tumor cells [8]. ctDNA contains the same genetic defects as the tumor DNA it originates from, e.g., point mutations, rearrangements, and amplifications [9], and can reflect the dynamic changes of tumors in real time with a short half-life in blood [10]. In addition, as a method for liquid biopsy, ctDNA detection can overcome the defects resulting from tumor heterogeneity in tissue biopsy and ensure more comprehensive detection [11]. Thus, the ctDNA detection can be used for early diagnosis and staging of cancer, tumor efficacy evaluation, tumor recurrence monitoring, and prognosis evaluation [12, 13]. Circulating microRNAs (miRNAs) are a class of endogenous non-coding small-molecule single-stranded RNAs sized approximately 18–24 nucleotides in length; these miRNAs regulate the expression of approximately 30% of human proteins and participate in the regulation of cell differentiation, growth, apoptosis, and metabolism. Recent studies have shown that miRNAs have specific expression profiles in various tumor tissues and that they are associated with various stages of tumorigenesis, tumor development, and metastasis [14]. At each stage, the corresponding miRNAs change along with the genes they regulate [15]. Long non-coding RNAs (lncRNAs) are a new type of non-coding RNA with a length of more than 200 nucleotides. lncRNAs are characterized by no or limited protein coding potential; further, they regulate gene expression at various levels in the form of RNA, for example via epigenetic regulation, transcriptional regulation, and post-transcriptional regulation [16]. Studies have confirmed that lncRNAs are involved in almost all types of biological processes, such as innate immunity, development, and tumorigenesis [17, 18]. In particular, the function of lncRNAs in cancer has widely been explored. Several researchers have reported that lncRNAs participate in carcinogenesis by regulating cell proliferation, division, differentiation, and metastasis [19, 20]. Furthermore, many studies have reported the potential of ctDNA, miRNAs, and lncRNAs as novel diagnostic molecular biomarkers for OC [10, 21, 22]. However, these published studies are inconsistent, and to the best of our knowledge, no preceding meta-analysis exists in the literature evaluating these three molecular biomarkers simultaneously.

We included three meta-analysis methods using different molecular signatures in this study. In these three meta-analyses, we aim to evaluate the diagnostic values of these different molecular signatures in OC, and particularly to analyze the discriminative value of ctDNA, miRNAs, and lncRNAs between OC patients and healthy controls.

## Methods

### Literature research strategy

These three meta-analyses were conducted in accordance with the PRISMA guidelines (S1 Checklist) [23]. We searched the PubMed, EMBASE, Cochrane Library, and Web of Science databases for all related articles published from January 1, 2015 until March 20, 2020. A large number of meta-analyses have been published before 2015 to evaluate the diagnostic value of ctDNA/miRNAs/lncRNAs [24, 25], but there is a lack of meta-analysis to evaluate these biomarkers simultaneously during 2015–2020. Thus, we restricted the start date of publications to January 1, 2015 for an updated and comprehensive meta-analysis. The search keywords were as follows: “Circulating Tumor DNA/MicroRNAs/RNA, Long Noncoding,” “Ovarian Neoplasms,” “early.” There were no language restrictions, but only English articles were included. If the title or abstract met the inclusion criteria, the full text was then evaluated for further verification.

### Inclusion and exclusion criteria

The inclusion criteria were as follows: (1) the diagnosis of OC patients was based on the histopathological analysis as the gold standard; (2) diagnostic tests used ctDNA/miRNAs/lncRNAs as molecular biomarkers for diagnosing OC; (3) the study was designed as a case-control study, with healthy individuals and patients with benign diseases included in the control group; (4) the study contained enough data to construct 2 × 2 diagnostic tables; (5) the sample size was more than 5 OC patients to reduce selection bias; and (6) the full text was published in English.

The following studies were excluded: (1) fraud studies; (2) descriptive studies wherein only the diagnostic value of ctDNA/miRNAs/lncRNAs in the diagnosis of OC was described without control groups; (3) studies with incorrect calculations and incomplete data; (4) conferences, reviews, abstracts, editorials, and case reports; and (5) duplicate data or duplicate publications. In addition, when several studies used the same patient cohort, only the latest, largest, or best quality studies was included.

### Data extraction and quality assessment

Two reviewers independently assessed the eligibility of the retrieved articles. Differences between reviewers were resolved by consulting with a third reviewer. The data characteristics of each article were as follows: last name of the first author (if the last name and the publication year of two articles were the same, the articles were arranged according to the last name of the first corresponding author), publication year, country of origin, specimen types, detection methods, pathology type, case and control numbers, biomarker types (ctDNA/miRNAs/lncRNAs), AUC and data of 2 × 2 diagnostic tables (including sensitivity and specificity). The methodological quality assessment of the articles included in this study was conducted as per the quality assessment of the diagnostic accuracy study-2 (QUADAS-2) [26]. QUADAS-2 contains four key domains: patient selection, index test, reference standard, and flow and timing, with “low,” “high,” or “unknown” as the results for the risk of bias. The QUADAS-2 quality evaluation chart was generated using Review Manager 5.3.3 (Cochrane collaboration, Barcelona, Spain).

### Statistical analysis

All analyses were performed using the statistical analysis software Meta Disc 1.4 (Cochrane Colloquium, Barcelona, Spain) and Stata 15.1 (Stata Corporation, College Station, USA). The sensitivity, specificity, diagnostic odds ratio (DOR), positive likelihood ratio (PLR), and negative likelihood ratio (NLR) of ctDNA/miRNAs/lncRNAs in OC diagnosis were analyzed using the bivariate meta-analysis model (BRM). The bivariate summary ROC (SROC) curve was generated by graphing the sensitivity and specificity of each included study. AUC values of 0.5–0.7, 0.7–0.9, and 0.9–1.0 indicate that the diagnostic accuracy is low, medium, and high, respectively. Additionally, Fagan nomograms and likelihood ratio plots were used to detect the clinical value of ctDNA/miRNAs/lncRNAs in OC diagnosis; in the former, a pre-test probability of 20% was assumed and then the post-test probability was calculated using Bayes’ theorem [27]. The spearman correlation coefficient, calculated using the logarithm of sensitivity and the logarithm of (1-specificity), was used to detect the threshold effect of the included studies. If threshold effect exists, a positive correlation appears. A spearman correlation coefficient of greater than 0.6 and a P value of less than 0.05 indicated that the threshold effect was statistically significant [28]. Heterogeneity between studies was evaluated using Cochran’s Q statistic and I^2^ statistic, and a P value of less than 0.1 or I^2^ value higher than 50% indicated significant heterogeneity [29, 30]. Random effect models were applied for calculating the pooled effect when obvious heterogeneity was observed. Otherwise, fixed effect models were chosen for calculating effect values [31]. Heterogeneity was further validated by subgroup and sensitivity analyses, and the source of heterogeneity was analyzed via single factor meta-regression. In our meta-analysis, because of heterogeneity, all statistical data were calculated using a random effects model. Further, Deeks’ funnel plots were used to measure the publication bias in the three meta-analyses, and a p value of less than 0.05 indicated a statistical publication bias [32].

## Results

### Search results

**Fig 1:**
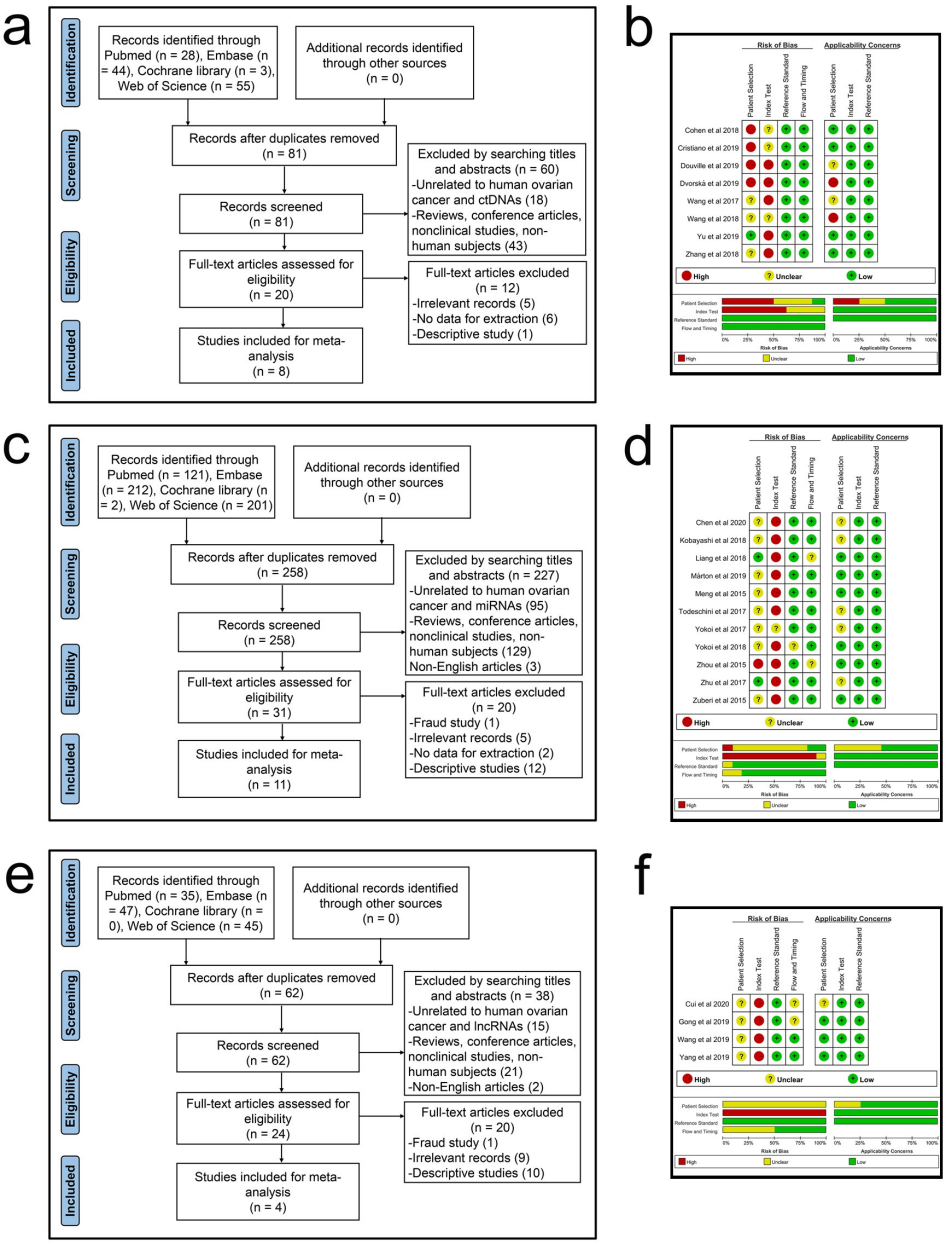
Flowcharts of literature search and identification and graphs of risk of bias and applicability concerns. (a) A flowchart of ctDNA. (b) A graph of bias risk and applicability concerns of ctDNA. (c) A flowchart of miRNAs. (d) A graph of bias risk and applicability concerns of miRNAs. (e) A flowchart of lncRNAs. (f) A graph of bias risk and applicability concerns of lncRNAs.

The literature screening process of ctDNA/miRNAs/lncRNAs is shown in Fig 1a, 1c and 1e, respectively. In all, 793 records (ctDNA: 130; miRNAs: 536; lncRNAs: 127) were initially identified on computer literature search, of which 392 duplicates (ctDNA: 49; miRNA: 278; lncRNA: 65) were excluded. After carefully evaluating the title, abstract, and keywords, 326 articles (ctDNA: 61; miRNA: 227; lncRNA: 38) were excluded because they were either reviews, conference articles, non-clinical trials, or non-human studies (ctDNA: 43; miRNA: 129; lncRNA: 21) or were not related to human OC and ctDNA/miRNAs/lncRNAs (ctDNA: 18; miRNA: 95; lncRNA: 15). Subsequently, full text of 75 articles (ctDNA: 20; miRNAs: 31; lncRNAs: 24) was obtained for further detailed review, and 52 of these (ctDNA: 12; miRNAs: 20; lncRNAs: 20) were excluded because they were fraud studies (miRNA: 1; lncRNA: 1) or irrelevant reports that were not related to diagnosis (ctDNA: 5; miRNA: 5; lncRNA: 9) or descriptive studies had no quantitative analysis of the diagnostic value of ctDNA/miRNA/lncRNA for OC (ctDNA: 1; miRNA: 12; lncRNA: 10) or reported insufficient data for constructing 2 × 2 diagnostic tables (ctDNA: 6; miRNA: 2). Finally, 23 articles (ctDNA: 8; miRNAs: 11; lncRNAs: 4) were included in this diagnostic meta-analysis [33–55].

### Study characteristics and quality assessment

The characteristics of ctDNA/miRNAs/lncRNAs included in the study are shown in Table 1. The 23 articles (ctDNA: 8; miRNAs: 11; lncRNAs: 4) included 1732 OC patients and 3958 controls (ctDNA: OC patients, 361, controls, 1773; miRNAs: OC patients, 1253, controls, 2002; lncRNAs: OC patients, 118, controls, 183), and all OC patients were diagnosed with the gold standard of histopathological analysis. With regard to the origins of these studies, most of the studies were concentrated in China (ctDNA: 3; miRNAs: 4; lncRNAs: 4), and other studies in the United States of America (ctDNA: 4), Slovakia (ctDNA: 1), Japan (miRNAs: 3), Hungary (miRNAs: 1), Germany (miRNAs: 1), Italy (miRNAs: 1), and India (miRNAs: 1). Subsequently, while analyzing the accuracy of ctDNA/miRNAs/lncRNAs in diagnosing OC, most studies tended to use plasma as a specimen (ctDNA: 8; miRNAs: 1; lncRNAs: 2), followed by serum (miRNAs: 9), tissue (lncRNAs: 2), and urine (miRNAs: 1). With regard to tumor lymphadenopathy (TNM) classification, 2 studies focused on stage I-II (lncRNAs: 2), 1 study concentrated on stage III-IV (miRNA: 1), and 19 studies focused on stage I-IV (ctDNA: 7; miRNAs: 10; lncRNAs: 2).

**Table 1:** Characteristics of included studies, divided by ctDNA, miRNAs and lncRNAs.

Author	Year	Country	Sample	Method	Pathology type	Case (Stage)	Case (n)	Control (Type)	Control (n)	Biomarkers	AUC	Sensitivity	Specificity
ctDNA													
Cohen et al	2018	USA	Plasma	Multiplex-PCR And Sequencing	EOC	I-IV	54	HC	812	BRAF, CDKN2A, CTNNB1, KRAS, PIK3CA, TP53	-	0.9815	0.9914
Cristiano et al	2019	USA	Plasma	WGS	OC	I-IV	28	HC	215	ALK, APC, AR, CTNNB1, EGFR, ERBB4, FGFR3, HNF1A, KIT, PDGFRA, SKT11, TP53	0.99	0.8929	0.9535
Douville et al	2019	USA	Plasma	RealSeqS	EOC	I-III	48	HC	378	-	0.989	0.979	0.905
Dvorská et al	2019	Slovakia	Plasma	PCR And Pyrosequencing	OC	I-IV	33	HC	9	RASSF1, PTEN, CDH1, PAX1	0.822	0.91	0.56
Wang et al	2017	China	Plasma	MSP	EOC	I-IV	71	HC	123	OPCML, RUNX3, TFPI2	-	0.9014	0.9187
Wang et al	2018	USA	Plasma	Multiplex-PCR And Sequencing	OC	I-IV	83	HC	192	PIK3CA, PIK3R1, PTEN, TP53	-	0.4337	1
Yu et al	2019	China	Plasma	qRT-PCR(SYBR-Green)	OC	I-IV	20	HC	20	ALU	0.861	0.8	0.6
Zhang et al	2018	China	Plasma	qRT-PCR(SYBR-Green)	OC	-	24	HC+BOD	24	ALU-219	0.73	0.667	0.792
miRNAs													
Chen et al	2020	China	Serum	qRT-PCR (TaqMan)	EOC	I-IV	152	HC+BOD+BOT	107	miR-125b	0.73	0.76	0.416
Kobayashi et al	2018	Japan	Serum	qRT-PCR (TaqMan)	EOC	I-IV	70	HC	13	miR-1290	0.48	0.51	0.57
Liang et al	2018	China	Serum	qRT-PCR (SYBR Green)	OC	I-IV	101	HC+BOD	100	miR-183	0.77	0.752	0.8
Márton et al	2019	Hungary	plasma	qRT-PCR (SYBR Green)	EOC	I+III+IV	28	HC+NM	60	miR-200c	0.861	0.7143	0.8667
Meng et al	2015	Germany	Serum	qRT-PCR (TaqMan)	EOC	I-IV	180	HC	66	miR-429	0.845	0.594	0.955
Todeschini et al	2017	Italy	Serum	qRT-PCR (SYBR Green)	EOC (HGSOC)	III-IV	168	HC	65	miR-1246	0.893	0.87	0.77
Yokoi et al	2018	Japan	Serum	Microarrays	OC	I-IV	160	CF	1379	miR-4532	0.974	0.956	0.928
Yokoi et al	2017	Japan	Serum	qRT-PCR (TaqMan)	EOC	I-IV	155	HC	63	miR-142	0.847	0.706	0.884
Zhou et al	2015	China	Urine	qRT-PCR (TaqMan)	EOC (SOC)	I-IV	34	HC	25	miR-6076	0.693	0.925	0.576
Zhu et al	2017	China	Serum	qRT-PCR (TaqMan)	EOC	I-IV	135	BOD	54	miR-125b	0.737	0.756	0.685
Zuberi et al	2015	India	Serum	qRT-PCR (SYBR Green)	EOC	I-IV	70	CF	70	miR-200a	0.81	0.806	0.735
lncRNAs													
Cui et al	2020	China	Plasma	qRT-PCR (SYBR Green)	OC	I-II	17	HC	58	CASC11	0.88	0.765	0.81
Gong et al	2019	China	Plasma	qRT-PCR (SYBR Green)	OC	I-II	28	HC	54	MIR4435-2HG	0.786	0.786	0.889
Yang et al	2019	China	Tissues	qRT-PCR (SYBR Green)	OC	I-IV	32	CF	31	FLJ33360	0.793	0.844	0.71
Wang et al	2019	China	Tissues	qRT-PCR (SYBR Green)	OC	I-IV	41	CF	40	HAGLROS	0.751	0.83	0.7

ctDNA, circulating tumor DNA; miRNAs, circulating microRNAs; lncRNAs, long non-coding RNAs; USA, the United States of America; AUC, area under curve; PCR, polymerase chain reaction; WGS, whole-genome sequencing; RealSeqS, repetitive element aneuploidy sequencing system; MSP, methylation-specific polymerase chain reaction; qRT-PCR, real-time fluorescence quantitative polymerase chain reaction; OC, ovarian cancer; EOC, epithelial ovarian cancer; HGSOC, high grade serous ovarian carcinoma; SOC, serous ovarian carcinoma; HC, healthy control; BOD, benign ovarian diseases; BOT, borderline ovarian tumors; NM, non-malignant masses; CF, cancer free; CA125, serum carbohydrate antigen 125; HE4, human epididymis protein 4.

All eligible studies were published between 2015 and 2020. The QUADAS-2 summary diagram for ctDNA/miRNAs/lncRNAs is shown in Fig 1b, 1d and 1f. The results demonstrate that the overall quality of all the included studies was stable. However, two important issues emerged. One was that due to the design of case-control studies, patient selection in eighteen studies may increase the risk of bias and applicability concerns. The other was that only two studies had set thresholds in advance, which may lead to unknown risks of bias in related articles.

### Diagnostic accuracy of ctDNA/miRNAs/lncRNAs in OC

**Fig 2:**
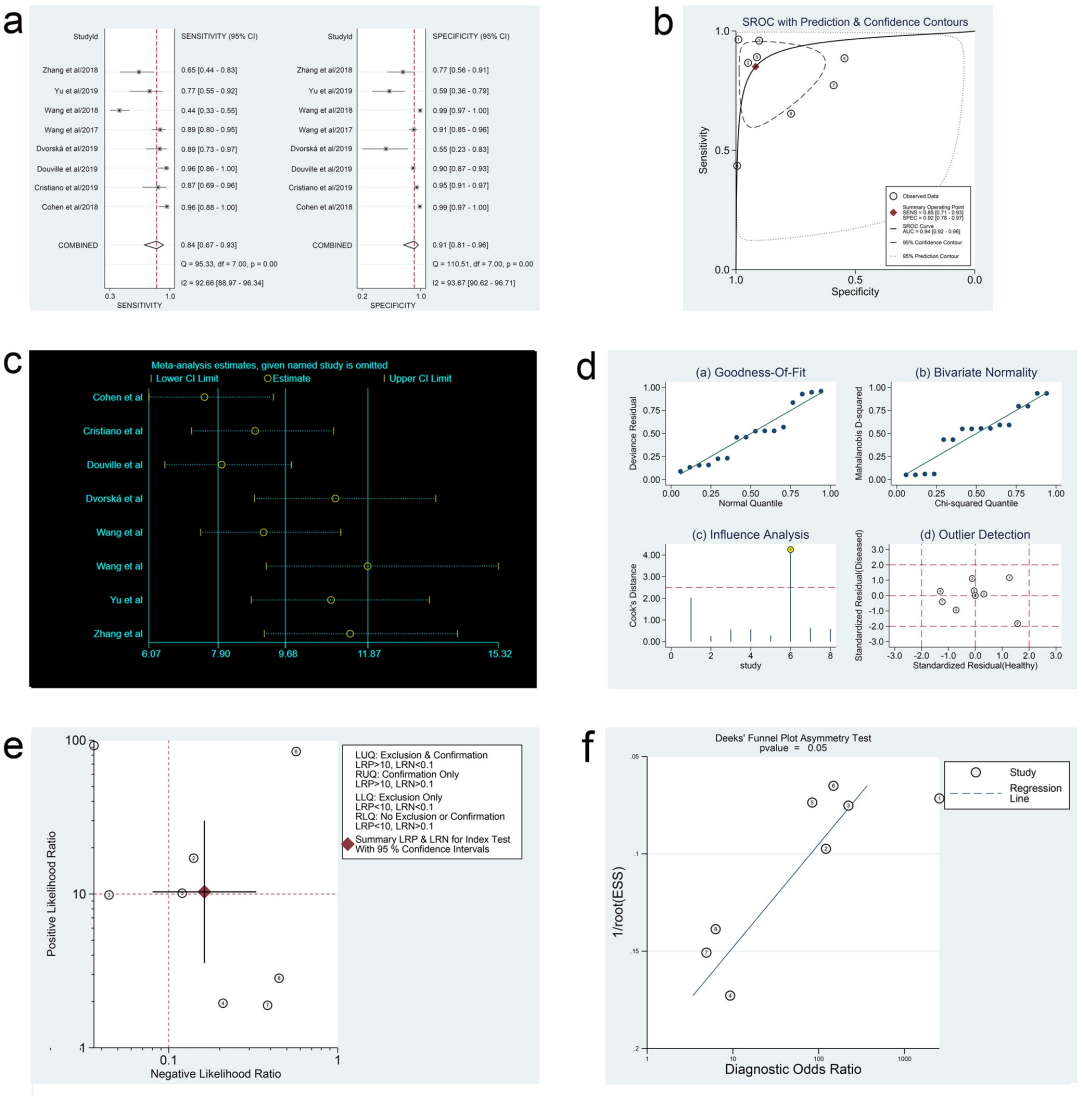
Forest plots of sensitivity and specificity, SROC curve, sensitivity analysis plot, goodness-of-fit and bivariate normality analysis plots, likelihood ratio plot and Deeks’ funnel plot of ctDNA. (a) Forest plots of sensitivity and specificity to evaluate the diagnostic performance of ctDNA. (b) SROC curve to describe the diagnostic value of ctDNA. (c) Sensitivity analysis to estimate each study’s value of ctDNA. (d) Goodness-of-fit and bivariate normality analysis plots to explore the sources of heterogeneity of ctDNA. (e) Likelihood ratio plot to appraise the diagnostic and elimination capabilities of ctDNA. (f) Deeks’ funnel plot to assess publication bias of ctDNA.

**Fig 3:**
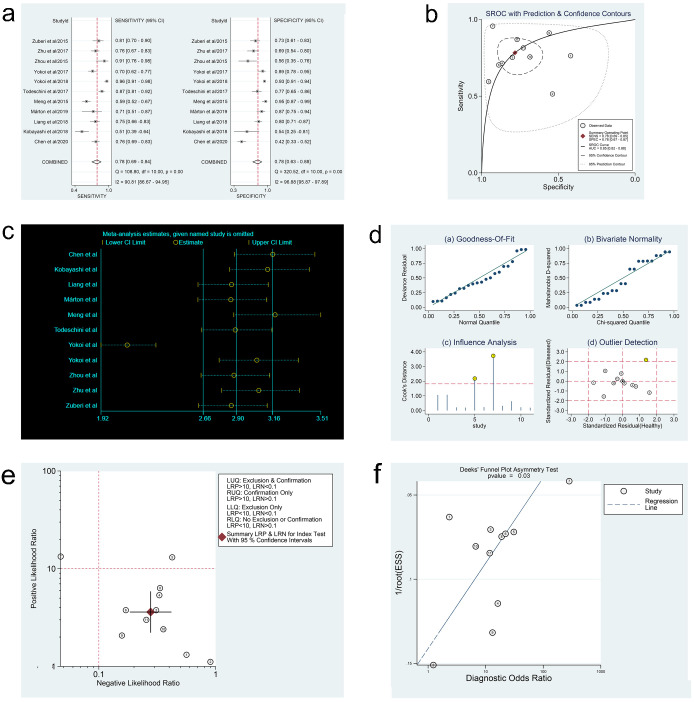
Forest plots of sensitivity and specificity, SROC curve, sensitivity analysis plot, goodness-of-fit and bivariate normality analysis plots, likelihood ratio plot and Deeks’ funnel plot of miRNAs. (a) Forest plots of sensitivity and specificity to evaluate the diagnostic performance of miRNAs. (b) SROC curve to describe the diagnostic value of miRNAs. (c) Sensitivity analysis to estimate each study’s value of miRNAs. (d) Goodness-of-fit and bivariate normality analysis plots to explore the sources of heterogeneity of miRNAs. (e) Likelihood ratio plot to appraise the diagnostic and elimination capabilities of miRNAs. (f) Deeks’ funnel plot to assess publication bias of miRNAs.

**Fig 4:**
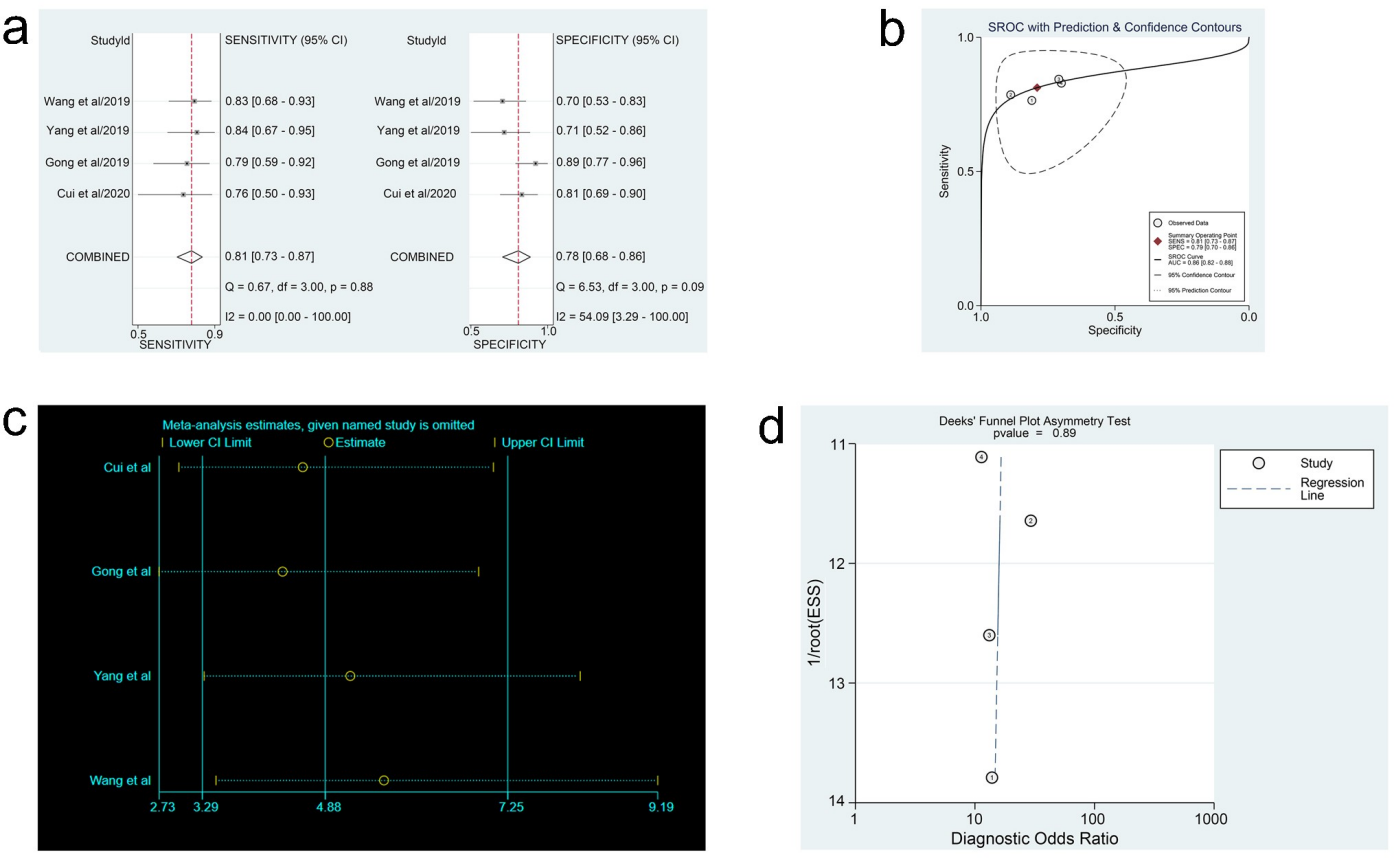
Forest plots of sensitivity and specificity, SROC curve, sensitivity analysis plot and Deeks’ funnel plot of lncRNAs. (a) Forest plots of sensitivity and specificity to evaluate the diagnostic performance of lncRNAs. (b) SROC curve to describe the diagnostic value of lncRNAs. (c) Sensitivity analysis to estimate each study’s value of lncRNAs. (d) Deeks’ funnel plot to assess publication bias of lncRNAs.

In the three meta-analyses, spearman correlation coefficients for ctDNA, miRNAs, and lncRNAs were -0.024 (P > 0.05), 0.118 (P > 0.05), and 0.600 (P > 0.05), respectively, which confirmed that there was no threshold effect and the heterogeneity was caused by other reasons in this study. Forest plots for sensitivity and specificity of ctDNA/miRNAs/lncRNAs in OC diagnosis are shown in Figs 2a, 3a and 4a. The heterogeneity caused by non-threshold effects was evaluated by Cochran’s Q statistic and I^2^ statistic, and I^2^ values for sensitivity and specificity of ctDNA/miRNAs/lncRNAs indicated heterogeneity caused by non-threshold effects (ctDNA: sensitivity, I^2^ = 89.70%, specificity, I^2^ = 68.09%; miRNAs: sensitivity, I^2^ = 88.30%, specificity, I^2^ = 95.50%; lncRNAs: sensitivity, I^2^ = 0.00%, specificity, I^2^ = 54.09%). The pooled sensitivity, specificity, PLR, NLR, AUC and DOR of ctDNA/miRNAs/lncRNAs for OC diagnosis is shown in Table 2. According to the SROC curves of the included studies, the AUC values indicating a high accuracy of ctDNA/miRNAs/lncRNAs in diagnosing OC (Figs 2b, 3b and 4b). The goodness-of-fit and bivariate normality analysis indicated that the random effects bivariate model was very suitable for the calculation of summary estimates (Figs 2d and 3d). Sensitivity analysis, influence analysis, and outlier detection analysis of ctDNA/miRNAs/lncRNAs revealed that the data reported in the record no. 6 of ctDNA, record no. 7 of miRNAs, and record no. 2 of lncRNAs are far from the rest of the data, suggesting that they could be one of the reasons for the heterogeneity (Figs 2c and 2d, 3c and 3d and 4c). After excluding record no. 6 of ctDNA, no. 7 of miRNAs, and no. 2 of lncRNAs, the random effect model was used to combine the effects. For ctDNA and miRNAs, the remaining data were still heterogeneous (ctDNA: I^2^ = 89.00%, P < 0.05; miRNAs: I^2^ = 82.20%, P < 0.05), and the I^2^ values for sensitivity and specificity were reduced by 16.88% and 0.75% for ctDNA and 5.27% and 6.64% for miRNAs, respectively (S1a and S1b Fig). The results for lncRNAs showed that the remaining 3 sets of data were not heterogeneous (I^2^ = 0.00, P > 0.1). This may be because of the superior diagnostic specificity of lncRNA MIR4435-2HG studied by Gong et al. compared with the remaining three markers (lncRNA CASC11, lncRNA FLJ33360, and lncRNA HAGLROS).

**Table 2:** Diagnostic efficacy of ctDNA, miRNAs and lncRNAs in OC.

	n	Sensitivity (95% CI)	Specificity (95% CI)	PLR (95% CI)	NLR (95% CI)	DOR (95% CI)	AUC (95% CI)
ctDNA	8	0.84 (0.67–0.93)	0.91 (0.81–0.96)	10.30 (3.60–29.80)	0.16 (0.08–0.33)	64.00 (16.00–247.00)	0.94 (0.92–0.96)
miRNAs	11	0.78 (0.69–0.84)	0.78 (0.63–0.88)	3.60 (2.20–5.80)	0.28 (0.19–0.42)	13.00 (6.00–29.00)	0.85 (0.82–0.88)
lncRNAs	4	0.81 (0.73–0.87)	0.78 (0.68–0.86)	3.62 (2.50–5.24)	0.25 (0.17–0.36)	15.30 (8.42–27.82)	0.86 (0.82–0.88)

ctDNA, circulating tumor DNA; miRNAs, circulating microRNAs; lncRNAs, long non-coding RNAs; CI, confidence interval; PLR, positive likelihood ratio; NLR, negative likelihood ratio; AUC, area under curve; DOR, diagnostic odds ratio.

### Meta-regression and subgroup analysis for heterogeneity

**Fig 5:**
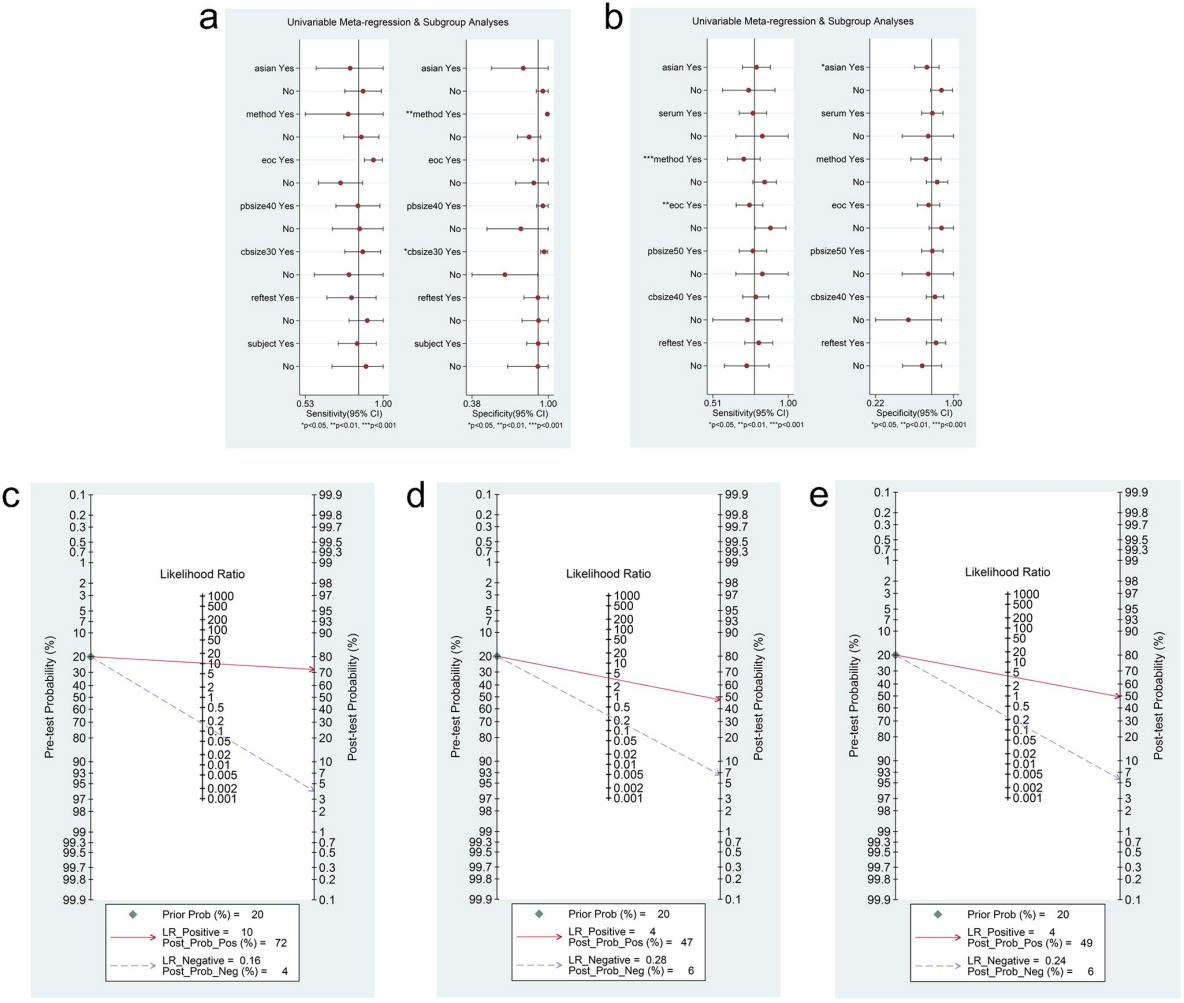
Meta-regression and Fagan’s Nomogram of ctDNA/miRNAs. (a) Meta-regression to explore heterogeneity between studies of ctDNA. (b) Meta-regression to explore heterogeneity between studies of miRNAs. (c) Fagan’s Nomogram to reveal the clinical application of ctDNA in the identification of OC patients and control individuals. (d) Fagan’s Nomogram to reveal the clinical application of miRNAs in the identification of OC patients and control individuals. (e) Fagan’s Nomogram to reveal the clinical application of lncRNAs in the identification of OC patients and control individuals.

To further explore the sources of heterogeneity, meta-regression analyses (Fig 5a and 5b) and subgroup analyses (Tables 3 and 4) were performed for ctDNA and miRNAs. For ctDNA, results of the meta-regression analysis depicted that methods (p = 0.00) and control size (p = 0.01) showed a statistically significant impact on specificity. Subgroup analysis indicated that ctDNA detection accuracy in the Caucasian population (DOR, 154.12; AUC, 0.97) showed a better diagnostic performance than that in the Asian population (DOR, 14.30; AUC,0.86). Subsequently, we noticed that ctDNA could detect all epithelial ovarian cancer (EOC) cases (DOR, 326.84; AUC, 0.98) more accurately than OC cases (DOR, 20.80; AUC, 0.86) on comparing pathology types. In addition, sample size of > 40 (DOR, 126.89; AUC, 0.97) showed superior diagnostic performance than that of ≤ 40 (DOR, 18.17; AUC, 0.91). Nevertheless, the subgroup based on control size suggested that a control size of > 30 (DOR, 219.93; AUC, 0.98) showed a better diagnostic performance than a control size of ≤ 30 (DOR, 6.34; AUC, 0.77). With regard to miRNAs, the results of the meta-regression analysis showed that methods of qRT-PCR (TaqMan) (p = 0.00) and case type (EOC cases or OC cases; p = 0.01) would affect the sensitivity. Further, ethnic differences (p = 0.03) significantly affected the specificity. Subgroup analysis revealed that the accuracy of detection using miRNAs in Asians (DOR, 10.98; AUC, 0.84) was inferior to that in Caucasians (DOR, 21.99; AUC, 0.89). In addition, it was remarkable that qRT-PCR (SYBR Green) (DOR, 14.87; AUC, 0.86), in contrast to qRT-PCR (TaqMan) (DOR, 7.04; AUC, 0.79), showed a better performance in predicting OC.

**Table 3:** Subgroup analysis of the diagnostic efficacy of ctDNA in OC.

	n	Sensitivity (95% CI)	Specificity (95% CI)	PLR (95% CI)	NLR (95% CI)	DOR (95% CI)	AUC	Heterogeneity (I^2^; P values)
Race								
Asian	3	0.82 (0.74–0.88)	0.85 (0.79–0.90)	3.80 (1.22–11.80)	0.27 (0.10–0.72)	14.30 (2.10–97.21)	0.86	(88%; 0.000)
Caucasian	5	0.77 (0.71–0.82)	0.95 (0.94–0.96)	16.43 (5.23–51.61)	0.13 (0.02–0.82)	154.12 (29.18–814.04)	0.97	(82%; 0.000)
Method								
Multiplex-PCR and sequencing	2	0.65 (NA)	0.99 (NA)	74.48 (NA)	0.36 (NA)	208.21 (NA)	NA	NA
Others	6	0.86 (0.81–0.91)	0.90 (0.88–0.92)	5.20 (2.48–10.89)	0.19 (0.09–0.40)	29.92 (8.06–111.09)	0.92	(85%; 0.000)
Pathology type								
EOC	3	0.93 (0.89–0.96)	0.94 (0.92–0.96)	19.02 (6.44–56.16)	0.07 (0.03–0.17)	326.84 (49.62–2152.93)	0.98	(83%; 0.003)
OC (Various types of OC)	5	0.65 (0.58–0.71)	0.93 (0.91–0.95)	5.65 (1.78–17.88)	0.35 (0.20–0.61)	20.80 (4.79–90.30)	0.86	(81%; 0.000)
Sample size								
≤ 40	3	0.85 (0.76–0.92)	0.90 (0.86–0.93)	3.98 (0.94–16.85)	0.23 (0.12–0.45)	18.17 (2.30–143.80)	0.91	(86%; 0.001)
> 40	5	0.76 (0.71–0.81)	0.95 (0.93–0.96)	15.37 (5.78–40.85)	0.15 (0.04–0.62)	126.89 (20.28–793.83)	0.97	(88%; 0.000)
Control size								
≤ 30	3	0.78 (0.68–0.87)	0.66 (0.53–0.78)	2.10 (1.45–3.04)	0.38 (0.25–0.59)	6.34 (2.92–13.79)	0.77	(0%; 0.830)
> 30	5	0.78 (0.73–0.83)	0.95 (0.94–0.96)	21.25 (9.59–47.07)	0.11 (0.02–0.65)	219.93 (73.95–654.03)	0.98	(67%; 0.017)

CI, confidence interval; PLR, positive likelihood ratio; NLR, negative likelihood ratio; AUC, area under curve; DOR, diagnostic odds ratio; PCR, polymerase chain reaction; NA, not available; EOC, epithelial ovarian cancer; OC, ovarian cancer.

**Table 4:** Subgroup analysis of the diagnostic efficacy of miRNAs in OC.

	n	Sensitivity (95% CI)	Specificity (95% CI)	PLR (95% CI)	NLR (95% CI)	DOR (95% CI)	AUC	Heterogeneity (I^2^; P values)
Race								
Asian	8	0.78 (0.75–0.80)	0.87 (0.85–0.88)	3.03 (1.36–6.73)	0.29 (0.17–0.50)	10.98 (3.51–34.36)	0.84	(94%; 0.000)
Caucasian	3	0.73 (0.68–0.77)	0.86 (0.81–0.91)	5.50 (2.93–10.34)	0.29 (0.15–0.57)	21.99 (12.77–37.86)	0.89	(0%; 0.729)
Specimen								
Serum	9	0.76 (0.73–0.78)	0.87 (0.85–0.89)	3.72 (1.75–7.89)	0.30 (0.20–0.47)	13.00 (4.72–35.76)	0.85	(94%; 0.000)
Plasma	1	0.71 (NA)	0.87 (NA)	5.36 (NA)	0.33 (NA)	16.26 (NA)	NA	NA
Urine	1	0.93 (NA)	0.58 (NA)	2.18 (NA)	0.13 (NA)	16.77 (NA)	NA	NA
Method								
qRT–PCR (TaqMan)	6	0.69 (0.66–0.72)	0.68 (0.62–0.73)	2.64 (1.38–5.05)	0.43 (0.33–0.56)	7.04 (2.71–18.26)	0.79	(86%; 0.000)
qRT–PCR (SYBR Green)	4	0.81 (0.77–0.85)	0.79 (0.74–0.83)	3.64 (2.90–4.56)	0.25 (0.19–0.35)	14.87 (10.04–22.01)	0.86	(0%; 0.606)
Microarrays	1	0.96 (NA)	0.93 (NA)	13.28 (NA)	0.05 (NA)	280.04 (NA)	NA	NA
Pathology type								
EOC	9	0.73 (0.70–0.76)	0.72 (0.68–0.76)	2.99 (1.83–4.88)	0.36 (0.27–0.47)	9.34 (4.55–19.15)	0.82	(84%; 0.000)
OC (Various types of OC)	2	0.88 (NA)	0.92 (NA)	10.87 (NA)	0.13 (NA)	81.32 (NA)	NA	NA
Sample size								
≤ 50	2	0.83 (NA)	0.78 (NA)	3.79 (NA)	0.22 (NA)	17.41 (NA)	NA	NA
> 50	9	0.76 (0.73–0.78)	0.87 (0.85–0.89)	3.72 (1.75–7.89)	0.30 (0.20–0.47)	13.00 (4.72–35.76)	0.85	(94%; 0.000)
Control size								
≤ 40	2	0.65 (NA)	0.57 (NA)	1.52 (NA)	0.62 (NA)	2.45 (NA)	NA	NA
> 40	9	0.77 (0.75–0.80)	0.87 (0.86–0.89)	4.41 (2.10–9.23)	0.28 (0.18–0.41)	16.93 (6.55–43.71)	0.87	(93%; 0.000)

CI, confidence interval; PLR, positive likelihood ratio; NLR, negative likelihood ratio; AUC, area under curve; DOR, diagnostic odds ratio; NA, not available; qRT-PCR, real-time fluorescence quantitative polymerase chain reaction; EOC, epithelial ovarian cancer; OC, ovarian cancer.

### Fagan nomogram analysis, likelihood ratio analysis and publication bias

Fagan’s nomogram plots were used to verify the probability of OC being detected by ctDNA/miRNAs/lncRNAs in an otherwise healthy person (Fig 5c–5e). For an individual with a 20% possibility of developing OC before the test, if the ctDNA, miRNAs, or lncRNAs are positive, the probability of being diagnosed with OC would reach 72%, 47%, or 49%, respectively. However, a negative ctDNA, miRNAs, or lncRNAs result implied that the probability after the test would reduce to 4%, 6%, or 6%, respectively. Accordingly, a PLR > 10 and NLR < 0.1 demonstrated high diagnostic accuracy [56]. In this study, the pooled PLR > 10 and NLR > 0.1 indicated that ctDNA showed a significantly high detection rate for OC, but exhibited a very low capacity for exclusion (Fig 2e). In other words, the use of ctDNA to diagnose OC may not be suitable as an exclusion test; however, ctDNA can be used as a confirmatory test. However, the pooled PLR < 10 and NLR > 0.1 of miRNAs indicated that based on the current research, the clinical application value of miRNAs in the diagnosis of OC is still limited and further studies are needed (Fig 3e). The potential publication bias of the included studies was evaluated using Deeks’ funnel plots, and its slope corresponded to p = 0.05 for ctDNA and p = 0.03 for miRNAs, suggesting that there is no publication bias for ctDNA in studies and a significant small publication bias for miRNA in studies (Figs 2f and 3f). Nevertheless, as only 4 articles were included for LncRNAs, although the Deeks’ funnel plot showed a slope corresponding to p = 0.89 (Fig 4d), there was still publication bias because of the small number of included articles.

## Discussion

OC is the main cause of gynecological malignant cancer-associated deaths, accounting for 4% of female malignant tumors worldwide [2, 5]. However, it has few obvious symptoms in the early stages, and most of patients are diagnosed in an advanced tumor stage, leading to high mortality rates in recent years [24]. Furthermore, there is a large discrepancy between the 5-year survival rates of early and late-stage patients, indicating the urgent need for new diagnostic biomarkers for OC.

Currently, the commonly used biomarkers for OC have low sensitivity or specificity. Zhen et al. studied the diagnostic value of serum CA125 and human epididymis protein 4 (HE4) in OC by constructing ROC curves (serum CA125: sensitivity, 0.74, specificity, 0.83; HE4: sensitivity, 0.74, specificity, 0.90), and reported low sensitivity of these two biomarkers [57]. The report by Guo et al. demonstrated that serum carbohydrate antigen 199 (CA199) had an appropriate diagnostic sensitivity (0.73) but low specificity (0.43) [58]. In addition, they also combined serum CA125 and serum CA199 for analysis, and sensitivity and specificity of this combination were 0.99 and 0.45, respectively, implying that serum CA199 and CA125 may not be suitable to distinguish between OC patients and normal individuals. Therefore, traditional diagnostic biomarkers for OC are not ideal for improving the diagnostic accuracy.

ctDNA originates from tumor cells and can reflect the dynamic changes of tumors in real time. miRNAs promote tumorigenesis by participating in the regulation of cell differentiation, growth, apoptosis, and metabolism. Further, lncRNAs participate in carcinogenesis by regulating cell proliferation, division, differentiation, and metastasis. Thus, they may have potential applications as biomarkers for the early detection of OC. However, the current studies focusing on the diagnostic value of ctDNA/miRNAs/lncRNAs in OC are inconsistent. Differences in specimen types (such as plasma or tissue), different inclusion criteria, and different detection techniques may explain the differences between different studies, but the lack of systematic evaluation complicates the conclusion. In addition, the understanding of the diversity value of ctDNA/miRNAs/lncRNAs is vague. Hence, we conducted this comprehensive and up-to-date study in a clinical context to further analyze the diagnostic value of ctDNA/miRNAs/lncRNAs.

In this comprehensive meta-analysis, we included 23 diagnostic studies to investigate whether ctDNA/miRNAs/lncRNAs are useful diagnostic molecular signatures for OC. We noted that ctDNA had high accuracy for OC diagnosis, with a pooled AUC of 0.94 (95% CI, 0.92–0.96), a pooled sensitivity of 0.84 (95% CI, 0.67–0.93), and a pooled specificity of 0.91 (95% CI, 0.81–0.96). The likelihood ratios (LRs) reflect the authenticity of sensitivity and specificity: the pooled PLR and NLR were 10.30 (95% CI, 3.60–29.80) and 0.16 (95% CI, 0.08–0.33), respectively, indicating that OC patients have an approximately 10 times greater chance of showing positive results in the ctDNA assay than healthy controls. When a true negative was detected in a ctDNA assay-negative test, the error rate was approximately 16%. The DOR, combining the advantages of sensitivity and specificity, is a significant indicator of diagnostic accuracy. The pooled DOR of ctDNA was 64.00 (95% CI, 16.00–247.00) in our study, implying that the diagnostic accuracy of ctDNA is high. Further, Fagan nomogram analysis and likelihood ratio analysis verified the higher diagnostic performance of ctDNA in diagnosing OC. We also found that lncRNAs were more accurate than miRNAs in diagnosing OC. The pooled AUC for lncRNAs was higher than that for miRNAs (0.86 vs. 0.85). In particular, the pooled sensitivity of lncRNAs was higher than that of miRNAs (0.81 vs. 0.78), and the specificities of these two biomarkers were quite similar (0.78 vs. 0.78). The likelihood ratio calculations confirmed that lncRNAs were similar to miRNAs in identifying OC (PLR, 3.62 vs. 3.60), whereas lncRNAs were superior to miRNAs in ruling out OC (NLR, 0.25 vs. 0.28). lncRNAs exhibited a higher pooled DOR compared with miRNAs (15.30 vs. 13.00). These results support the hypothesis that ctDNA/miRNAs/lncRNAs may be effective biomarkers for the diagnosis of OC. Additionally, combining these three biomarkers may help avoid the shortcomings of using a single diagnostic biomarker with insufficient sensitivity or specificity. These results also suggest the potential of combining ctDNA, miRNAs, and lncRNAs as diagnostic biomarkers for OC.

Heterogeneity is a significant research issue in meta-analyses. In this meta-analysis, Q-test and I^2^ statistical analysis revealed significant heterogeneity of the included studies. The threshold effect is the primary factor affecting the heterogeneity of diagnostic meta-analyses. In this study, the spearman correction coefficients of ctDNA/miRNAs/lncRNAs (ctDNA: -0.024, p > 0.05; miRNAs: 0.118, p > 0.05; lncRNAs: 0.600, p > 0.05) indicated that there was no threshold effect and the heterogeneity was caused by other reasons. To analyze the potential sources of heterogeneity, we used meta-regression and subgroup analysis for investigating the characteristics of the included studies, such as ethnic differences, sample types, case types, and detection methods among others. We noted that for ctDNA, ctDNA could be detected more accurately in the Caucasian population than in the Asian population. Subsequently, all EOC cases had a higher diagnostic accuracy of ctDNA for prediction, as compared to OC cases. In addition, a larger sample size showed a better diagnostic performance than a smaller sample size. As far as miRNAs are concerned, the accuracy of detection using miRNAs in Asians was inferior to that in Caucasians. Furthermore, the detection method of qRT-PCR (SYBR-Green) was verified to have a significantly better performance with regard to OC prediction compared with qRT-PCR (TaqMan). Publication bias was not significant for ctDNA, indicating that our meta-analysis results are reliable. However, the possible reasons for publication bias of miRNAs were as follows: (1) only English studies were included, indicating that language bias may be the source of publication bias; (2) the evaluation index of the included studies was the consistency of miRNA expression results and histopathological biopsy results, and there was a possibility that the authors preferred to publish positive results; (3) the small number of included cases may affect the accuracy of the statistical results. These results also indicate that the current evidence cannot determine the best sources of heterogeneity (ctDNA/miRNAs) for the reliable detection of OC. To confirm the findings of the current study, further large-scale studies with different ethnic groups, sample types, case types, detection methods, and sample sizes are required.

Our current research does have some limitations. First, ctDNA/miRNAs/lncRNAs are recently discovered tumor biomarkers; accordingly, the number of studies that could be included in the study was relatively small, resulting in poor stability of some pooled analysis results. This issue can be improved when more research data on these markers are available. In addition, the included studies lack CA125 levels, so it cannot be ruled out whether CA125 testing can complement the performance of accuracy of ctDNA/miRNAs/lncRNAs alone or in combination. Besides, 11 of the 23 eligible studies were conducted in China, which may result in a selection bias for specific study populations. Although we conducted subgroup analyses to find the source of heterogeneity, we could not fully explain the heterogeneity. Furthermore, some data were calculated based on the data extracted from ROC curves, which may not be as powerful as the data obtained directly from articles.

Despite these limitations, our meta-analysis has several important advantages. First, this was a relatively comprehensive systematic study, wherein the diagnostic value of ctDNA/miRNAs/lncRNAs in OC was independently estimated and verified. Furthermore, our method was strict and followed the guidelines for conducting and reporting systematic reviews.

## Conclusion

In summary, the current evidence indicates that ctDNA/miRNAs/lncRNAs are highly correlated with OC, and may be potential and promising biomarkers for distinguishing OC patients from healthy controls. Further large-scale studies are needed to verify the potential applicability of ctDNA/miRNAs/lncRNAs alone or in combination as OC diagnostic molecular biomarkers and to explore the potential factors that may affect the accuracy of ctDNA/miRNAs/lncRNAs in OC diagnosis.

## Supporting information

S1 Checklist PRISMA 2009 checklist. (DOC)

S1 Fig Forest plots for studies on ctDNA/miRNAs used in the diagnosis of OC among records (outlier records were excluded). (a) Forest plots of sensitivity and specificity of ctDNA after excluding outlier record. (b) Forest plots of sensitivity and specificity of miRNAs after excluding outlier record.
(TIF)

S1 File The search strategy of ctDNA, miRNAs and lncRNAs. (DOCX)
