# Supplementary figures and images for: In Silico screening of circulating tumor DNA, circulating microRNAs, and long non-coding RNAs as diagnostic molecular biomarkers in ovarian cancer: A comprehensive meta-analysis
Source: PLoS One. 2021 Apr 26;16(4):e0250717. doi: 10.1371/journal.pone.0250717 (PMC8075214; doi:10.1371/journal.pone.0250717)

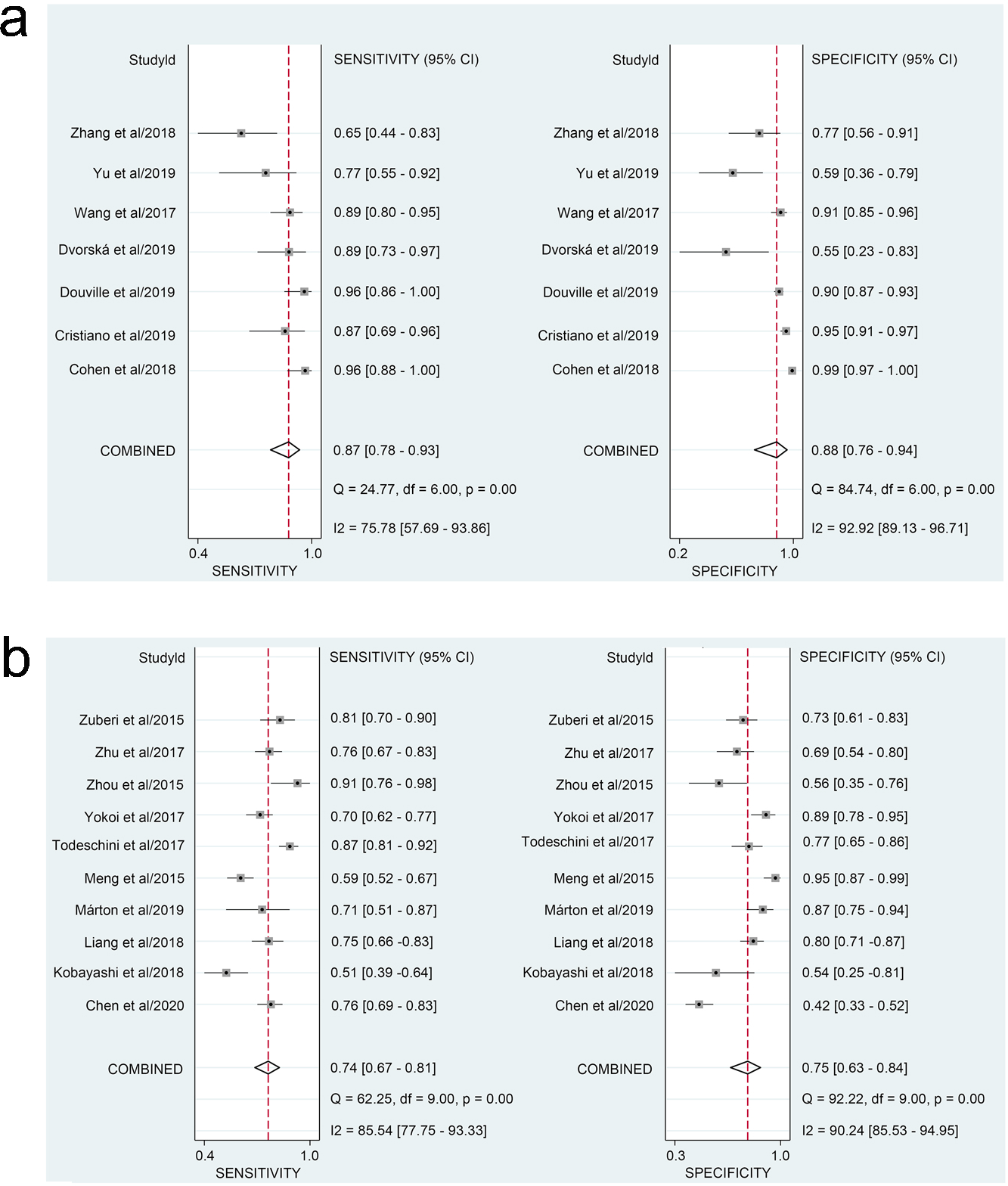

Supplement: S1 Fig — (a) Forest plots of sensitivity and specificity of ctDNA after excluding outlier record. (b) Forest plots of sensitivity and specificity of miRNAs after excluding outlier record. (TIF) [file pone.0250717.s002.tif]
