# Supplementary material for: In Silico screening of circulating tumor DNA, circulating microRNAs, and long non-coding RNAs as diagnostic molecular biomarkers in ovarian cancer: A comprehensive meta-analysis
Source: PLoS One. 2021 Apr 26;16(4):e0250717. doi: 10.1371/journal.pone.0250717 (PMC8075214; doi:10.1371/journal.pone.0250717)
Supplement: S1 File — (DOCX) [file pone.0250717.s003.docx]

***In Silico* Screening of Circulating Tumor DNA, Circulating MicroRNAs and Long Non-coding RNAs as Diagnostic Molecular Biomarkers in Ovarian Cancer: a comprehensive meta-analysis**

**Search strategy**

**Circulating Tumor DNA (ctDNA) search strategy:**

Pubmed search strategy: 28

Search (((((("Ovarian Neoplasms"[Mesh] AND ( "2015/01/01"[PDat] : "2020/03/20"[PDat] ))) OR ((((((((((((((((((Neoplasm, Ovarian[Title/Abstract]) OR Ovarian Neoplasm[Title/Abstract]) OR Ovary Neoplasms[Title/Abstract]) OR Neoplasm, Ovary[Title/Abstract]) OR Neoplasms, Ovary[Title/Abstract]) OR Ovary Neoplasm[Title/Abstract]) OR Neoplasms, Ovarian[Title/Abstract]) OR Ovary Cancer[Title/Abstract]) OR Cancer, Ovary[Title/Abstract]) OR Cancers, Ovary[Title/Abstract]) OR Ovary Cancers[Title/Abstract]) OR Ovarian Cancer[Title/Abstract]) OR Cancer, Ovarian[Title/Abstract]) OR Cancers, Ovarian[Title/Abstract]) OR Ovarian Cancers[Title/Abstract]) OR Cancer of Ovary[Title/Abstract]) OR Cancer of the Ovary[Title/Abstract]) AND ( "2015/01/01"[PDat] : "2020/03/20"[PDat] ))) AND ( "2015/01/01"[PDat] : "2020/03/20"[PDat] ))) AND (((("Circulating Tumor DNA"[Mesh] AND ( "2015/01/01"[PDat] : "2020/03/20"[PDat] ))) OR (((((((DNA, Circulating Tumor[Title/Abstract]) OR Tumor DNA, Circulating[Title/Abstract]) OR Cell-Free Tumor DNA[Title/Abstract]) OR Cell Free Tumor DNA[Title/Abstract]) OR DNA, Cell-Free Tumor[Title/Abstract]) OR Tumor DNA, Cell-Free[Title/Abstract]) AND ( "2015/01/01"[PDat] : "2020/03/20"[PDat] ))) AND ( "2015/01/01"[PDat] : "2020/03/20"[PDat] ))) AND early[Title/Abstract] Filters: Publication date from 2015/01/01 to 2020/03/20

Embase search strategy: 44


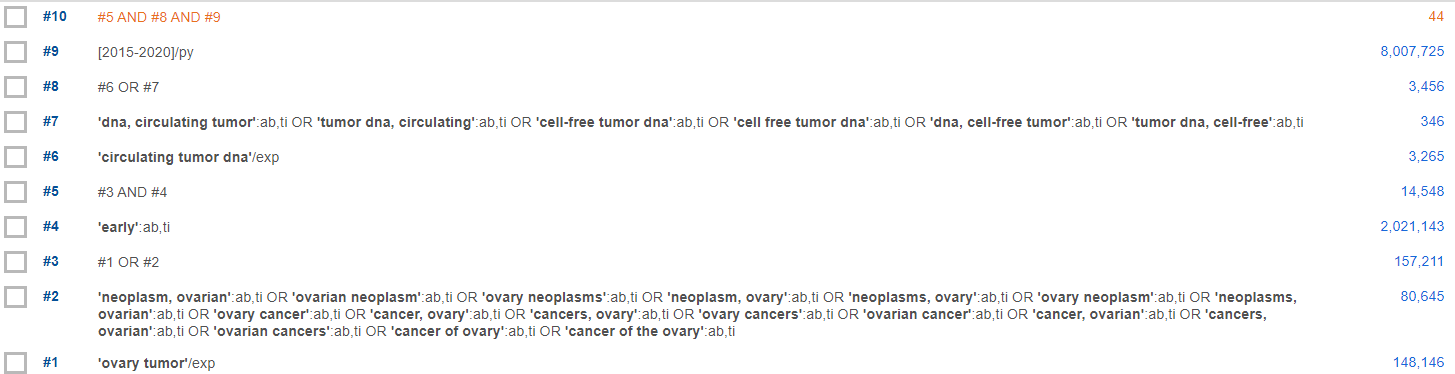


Cochrane search strategy: 3


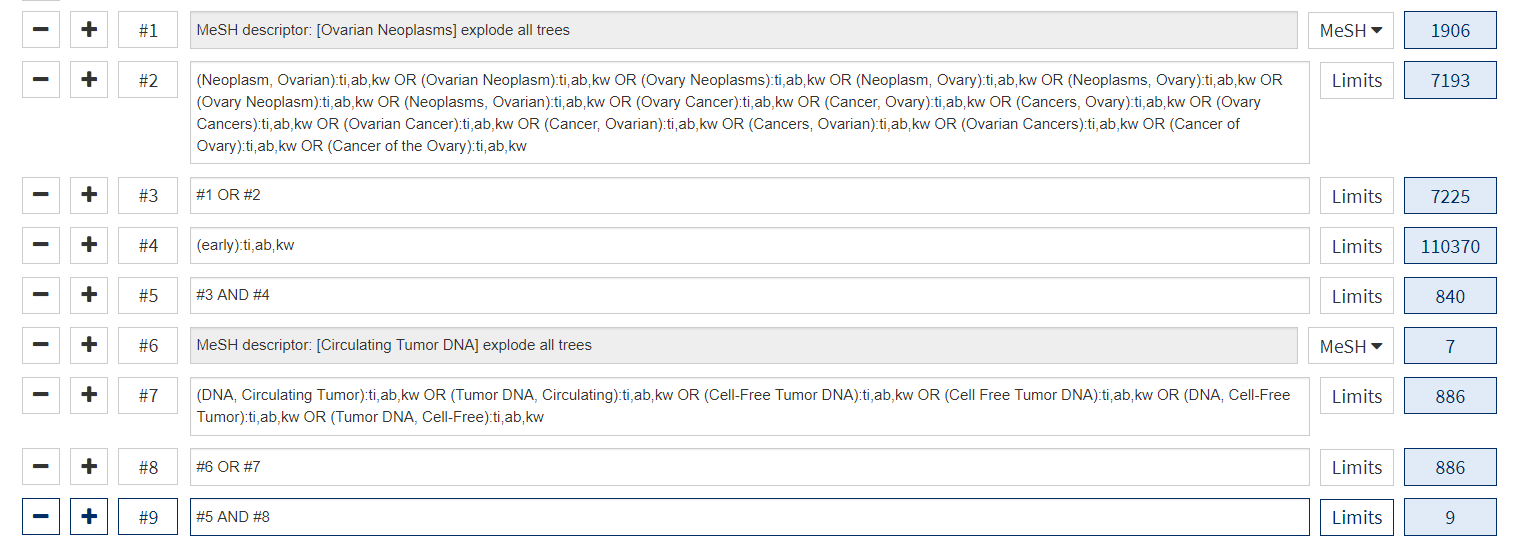


Web of Science search strategy: 55

| Set | Results | save History / Create Alert Open Saved History |
| --- | --- | --- |
| # 4 | [55](https://apps.webofknowledge.com/summary.do?product=WOS&doc=1&qid=111105&SID=D2M389uhNRJFzFfqRpG&search_mode=CombineSearches&update_back2search_link_param=yes) | #3 AND #2 AND #1  索引=SCI-EXPANDED, SSCI, A&HCI, CPCI-S, CPCI-SSH, BKCI-S, BKCI-SSH, ESCI, CCR-EXPANDED, IC 时间跨度=2015-2020 |
| # 3 | [7,626](https://apps.webofknowledge.com/summary.do?product=WOS&doc=1&qid=111104&SID=D2M389uhNRJFzFfqRpG&search_mode=AdvancedSearch&update_back2search_link_param=yes) | TS=(Circulating Tumor DNA OR DNA, Circulating Tumor OR Tumor DNA, Circulating OR Cell-Free Tumor DNA OR Cell Free Tumor DNA OR DNA, Cell-Free Tumor OR Tumor DNA, Cell-Free)  索引=SCI-EXPANDED, SSCI, A&HCI, CPCI-S, CPCI-SSH, BKCI-S, BKCI-SSH, ESCI, CCR-EXPANDED, IC 时间跨度=2015-2020 |
| # 2 | [752,393](https://apps.webofknowledge.com/summary.do?product=WOS&doc=1&qid=111087&SID=D2M389uhNRJFzFfqRpG&search_mode=AdvancedSearch&update_back2search_link_param=yes) | TS=(early)  索引=SCI-EXPANDED, SSCI, A&HCI, CPCI-S, CPCI-SSH, BKCI-S, BKCI-SSH, ESCI, CCR-EXPANDED, IC 时间跨度=2015-2020 |
| # 1 | [40,656](https://apps.webofknowledge.com/summary.do?product=WOS&doc=1&qid=111086&SID=D2M389uhNRJFzFfqRpG&search_mode=AdvancedSearch&update_back2search_link_param=yes) | TS=(Ovarian Neoplasms OR Neoplasm, Ovarian OR Ovarian Neoplasm OR Ovary Neoplasms OR Neoplasm, Ovary OR Neoplasms, Ovary OR Ovary Neoplasm OR Neoplasms, Ovarian OR Ovary Cance OR Cancer, Ovary OR Cancers, Ovary OR Ovary Cancers OR Ovarian Cancer OR Cancer, Ovarian OR Cancers, Ovarian OR Ovarian Cancers OR Cancer of Ovary OR Cancer of the Ovary)  索引=SCI-EXPANDED, SSCI, A&HCI, CPCI-S, CPCI-SSH, BKCI-S, BKCI-SSH, ESCI, CCR-EXPANDED, IC 时间跨度=2015-2020 |

**Circulating MicroRNAs (miRNAs) search strategy:**

Pubmed search strategy: 121

Search (((("Ovarian Neoplasms"[Mesh]) OR (((((((((((((((((Neoplasm, Ovarian[Title/Abstract]) OR Ovarian Neoplasm[Title/Abstract]) OR Ovary Neoplasms[Title/Abstract]) OR Neoplasm, Ovary[Title/Abstract]) OR Neoplasms, Ovary[Title/Abstract]) OR Ovary Neoplasm[Title/Abstract]) OR Neoplasms, Ovarian[Title/Abstract]) OR Ovary Cancer[Title/Abstract]) OR Cancer, Ovary[Title/Abstract]) OR Cancers, Ovary[Title/Abstract]) OR Ovary Cancers[Title/Abstract]) OR Ovarian Cancer[Title/Abstract]) OR Cancer, Ovarian[Title/Abstract]) OR Cancers, Ovarian[Title/Abstract]) OR Ovarian Cancers[Title/Abstract]) OR Cancer of Ovary[Title/Abstract]) OR Cancer of the Ovary[Title/Abstract]))) AND (("MicroRNAs"[Mesh]) OR ((((((MicroRNA[Title/Abstract]) OR miRNAs[Title/Abstract]) OR Micro RNA[Title/Abstract]) OR RNA, Micro[Title/Abstract]) OR miRNA[Title/Abstract]) OR miR[Title/Abstract]))) AND early[Title/Abstract] Filters: Publication date from 2015/01/01 to 2020/03/20

Embase search strategy: 212


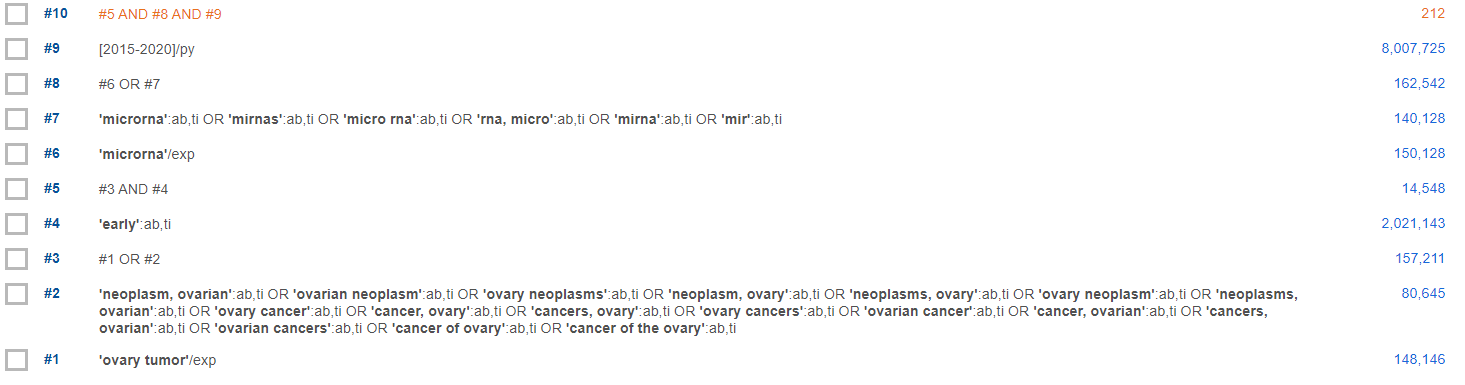


Cochrane search strategy: 2


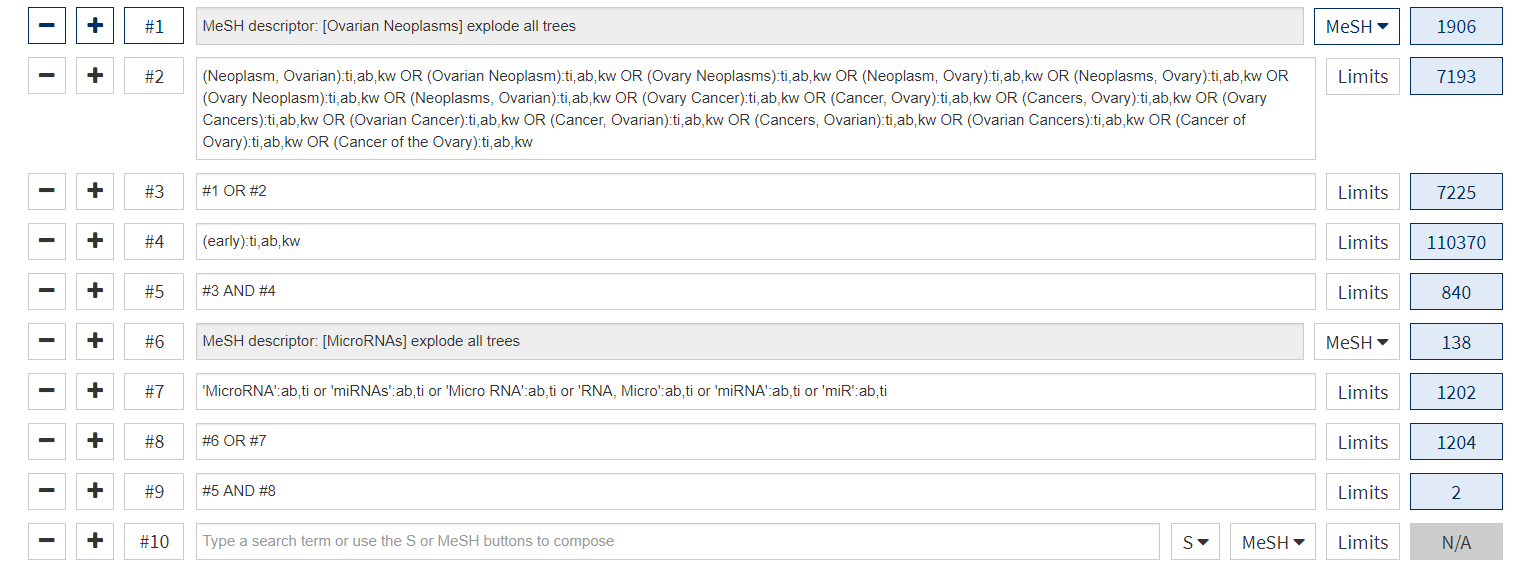


Web of Science search strategy: 201

| Set | Results | save History / Create Alert Open Saved History |
| --- | --- | --- |
| # 4 | [201](https://apps.webofknowledge.com/summary.do?product=WOS&doc=1&qid=111089&SID=D2M389uhNRJFzFfqRpG&search_mode=CombineSearches&update_back2search_link_param=yes) | #3 AND #2 AND #1  索引=SCI-EXPANDED, SSCI, A&HCI, CPCI-S, CPCI-SSH, BKCI-S, BKCI-SSH, ESCI, CCR-EXPANDED, IC 时间跨度=2015-2020 |
| # 3 | [89,896](https://apps.webofknowledge.com/summary.do?product=WOS&doc=1&qid=111088&SID=D2M389uhNRJFzFfqRpG&search_mode=AdvancedSearch&update_back2search_link_param=yes) | TS=(MicroRNAs OR MicroRNA OR miRNAs OR Micro RNA OR RNA, Micro OR miRNA OR miR)  索引=SCI-EXPANDED, SSCI, A&HCI, CPCI-S, CPCI-SSH, BKCI-S, BKCI-SSH, ESCI, CCR-EXPANDED, IC 时间跨度=2015-2020 |
| # 2 | [752,393](https://apps.webofknowledge.com/summary.do?product=WOS&doc=1&qid=111087&SID=D2M389uhNRJFzFfqRpG&search_mode=AdvancedSearch&update_back2search_link_param=yes) | TS=(early)  索引=SCI-EXPANDED, SSCI, A&HCI, CPCI-S, CPCI-SSH, BKCI-S, BKCI-SSH, ESCI, CCR-EXPANDED, IC 时间跨度=2015-2020 |
| # 1 | [40,656](https://apps.webofknowledge.com/summary.do?product=WOS&doc=1&qid=111086&SID=D2M389uhNRJFzFfqRpG&search_mode=AdvancedSearch&update_back2search_link_param=yes) | TS=(Ovarian Neoplasms OR Neoplasm, Ovarian OR Ovarian Neoplasm OR Ovary Neoplasms OR Neoplasm, Ovary OR Neoplasms, Ovary OR Ovary Neoplasm OR Neoplasms, Ovarian OR Ovary Cance OR Cancer, Ovary OR Cancers, Ovary OR Ovary Cancers OR Ovarian Cancer OR Cancer, Ovarian OR Cancers, Ovarian OR Ovarian Cancers OR Cancer of Ovary OR Cancer of the Ovary)  索引=SCI-EXPANDED, SSCI, A&HCI, CPCI-S, CPCI-SSH, BKCI-S, BKCI-SSH, ESCI, CCR-EXPANDED, IC 时间跨度=2015-2020 |

**Long Non-coding RNAs (lncRNAs) search strategy:**

Pubmed search strategy: 35

Search (((((("Ovarian Neoplasms"[Mesh] AND ( "2015/01/01"[PDat] : "2020/03/20"[PDat] ))) OR ((((((((((((((((((Neoplasm, Ovarian[Title/Abstract]) OR Ovarian Neoplasm[Title/Abstract]) OR Ovary Neoplasms[Title/Abstract]) OR Neoplasm, Ovary[Title/Abstract]) OR Neoplasms, Ovary[Title/Abstract]) OR Ovary Neoplasm[Title/Abstract]) OR Neoplasms, Ovarian[Title/Abstract]) OR Ovary Cancer[Title/Abstract]) OR Cancer, Ovary[Title/Abstract]) OR Cancers, Ovary[Title/Abstract]) OR Ovary Cancers[Title/Abstract]) OR Ovarian Cancer[Title/Abstract]) OR Cancer, Ovarian[Title/Abstract]) OR Cancers, Ovarian[Title/Abstract]) OR Ovarian Cancers[Title/Abstract]) OR Cancer of Ovary[Title/Abstract]) OR Cancer of the Ovary[Title/Abstract]) AND ( "2015/01/01"[PDat] : "2020/03/20"[PDat] ))) AND ( "2015/01/01"[PDat] : "2020/03/20"[PDat] ))) AND (((("RNA, Long Noncoding"[Mesh] AND ( "2015/01/01"[PDat] : "2020/03/20"[PDat] ))) OR (((((((((((((((((((((((((((Noncoding RNA, Long[Title/Abstract]) OR lncRNA[Title/Abstract]) OR Long ncRNA[Title/Abstract]) OR ncRNA, Long[Title/Abstract]) OR RNA, Long Non-Translated[Title/Abstract]) OR Long Non-Translated RNA[Title/Abstract]) OR Non-Translated RNA, Long[Title/Abstract]) OR RNA, Long Non Translated[Title/Abstract]) OR Long Non-Coding RNA[Title/Abstract]) OR Long Non Coding RNA[Title/Abstract]) OR Non-Coding RNA, Long[Title/Abstract]) OR RNA, Long Non-Coding[Title/Abstract]) OR Long Non-Protein-Coding RNA[Title/Abstract]) OR Long Non Protein Coding RNA[Title/Abstract]) OR Non-Protein-Coding RNA, Long[Title/Abstract]) OR RNA, Long Non-Protein-Coding[Title/Abstract]) OR Long Noncoding RNA[Title/Abstract]) OR RNA, Long Untranslated[Title/Abstract]) OR Long Untranslated RNA[Title/Abstract]) OR Untranslated RNA, Long[Title/Abstract]) OR Long ncRNAs[Title/Abstract]) OR ncRNAs, Long[Title/Abstract]) OR Long Intergenic Non-Protein Coding RNA[Title/Abstract]) OR Long Intergenic Non Protein Coding RNA[Title/Abstract]) OR LincRNAs[Title/Abstract]) OR LINC RNA[Title/Abstract]) AND ( "2015/01/01"[PDat] : "2020/03/20"[PDat] ))) AND ( "2015/01/01"[PDat] : "2020/03/20"[PDat] ))) AND early[Title/Abstract] Filters: Publication date from 2015/01/01 to 2020/03/20

Embase search strategy: 47


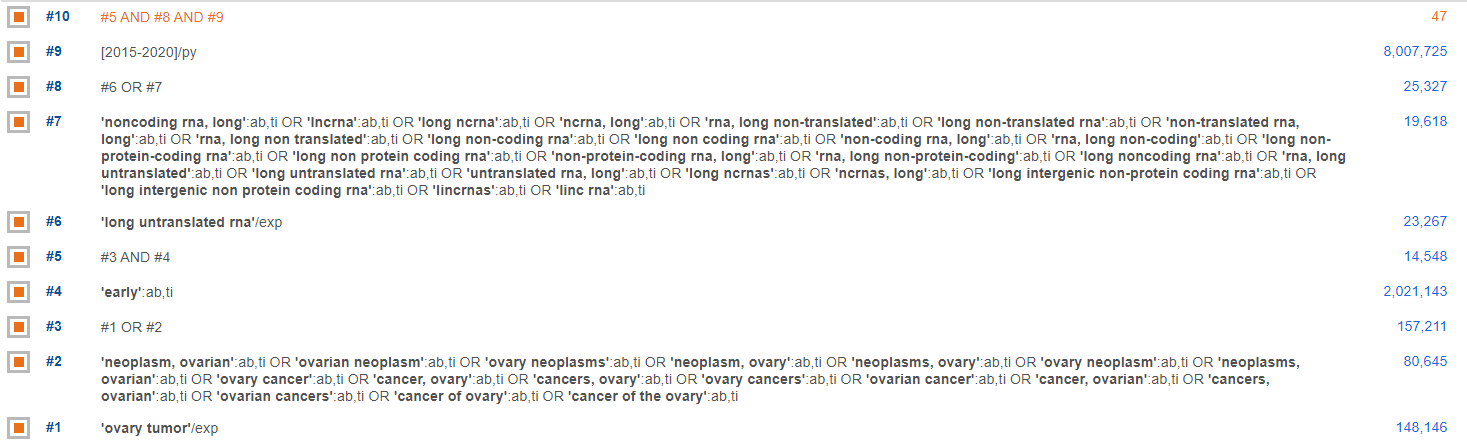


Cochrane search strategy: 0


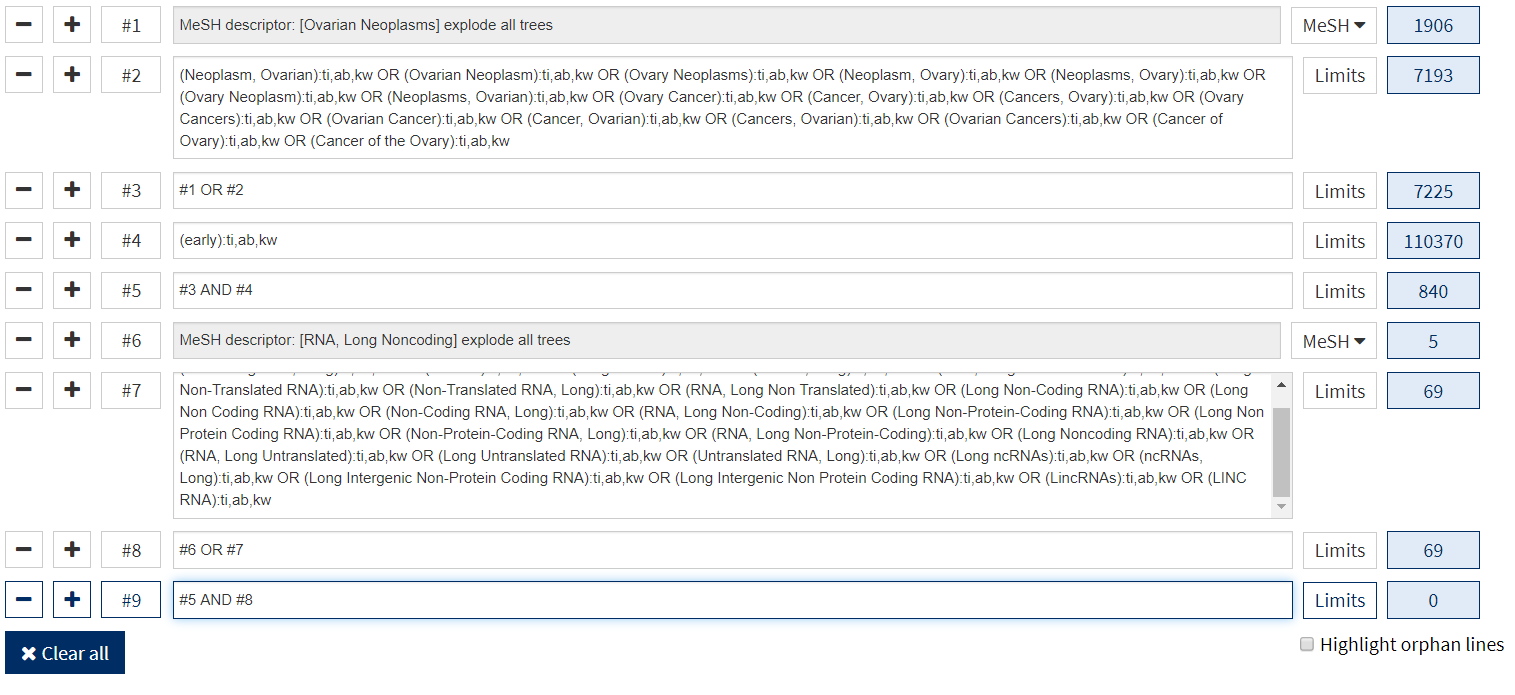


Web of Science search strategy: 45

| Set | Results | save History / Create Alert Open Saved History |
| --- | --- | --- |
| # 4 | [45](https://apps.webofknowledge.com/summary.do?product=WOS&doc=1&qid=111093&SID=D2M389uhNRJFzFfqRpG&search_mode=CombineSearches&update_back2search_link_param=yes) | #3 AND #2 AND #1  索引=SCI-EXPANDED, SSCI, A&HCI, CPCI-S, CPCI-SSH, BKCI-S, BKCI-SSH, ESCI, CCR-EXPANDED, IC 时间跨度=2015-2020 |
| # 3 | [22,476](https://apps.webofknowledge.com/summary.do?product=WOS&doc=1&qid=111092&SID=D2M389uhNRJFzFfqRpG&search_mode=AdvancedSearch&update_back2search_link_param=yes) | TS=(RNA, Long Noncoding OR Noncoding RNA, Long OR lncRNA OR Long ncRNA OR ncRNA, Long OR RNA, Long Non-Translated OR Long Non-Translated RNA OR Non-Translated RNA, Long OR RNA, Long Non Translated OR Long Non-Coding RNA OR Long Non Coding RNA OR Non-Coding RNA, Long OR RNA, Long Non-Coding OR Long Non-Protein-Coding RNA OR Long Non Protein Coding RNA OR Non-Protein-Coding RNA, Long OR RNA, Long Non-Protein-Coding OR Long Noncoding RNA OR RNA, Long Untranslated OR Long Untranslated RNA OR Untranslated RNA, Long OR Long ncRNAs OR ncRNAs, Long OR Long Intergenic Non-Protein Coding RNA OR Long Intergenic Non Protein Coding RNA OR LincRNAs OR LINC RNA)  索引=SCI-EXPANDED, SSCI, A&HCI, CPCI-S, CPCI-SSH, BKCI-S, BKCI-SSH, ESCI, CCR-EXPANDED, IC 时间跨度=2015-2020 |
| # 2 | [752,393](https://apps.webofknowledge.com/summary.do?product=WOS&doc=1&qid=111087&SID=D2M389uhNRJFzFfqRpG&search_mode=AdvancedSearch&update_back2search_link_param=yes) | TS=(early)  索引=SCI-EXPANDED, SSCI, A&HCI, CPCI-S, CPCI-SSH, BKCI-S, BKCI-SSH, ESCI, CCR-EXPANDED, IC 时间跨度=2015-2020 |
| # 1 | [40,656](https://apps.webofknowledge.com/summary.do?product=WOS&doc=1&qid=111086&SID=D2M389uhNRJFzFfqRpG&search_mode=AdvancedSearch&update_back2search_link_param=yes) | TS=(Ovarian Neoplasms OR Neoplasm, Ovarian OR Ovarian Neoplasm OR Ovary Neoplasms OR Neoplasm, Ovary OR Neoplasms, Ovary OR Ovary Neoplasm OR Neoplasms, Ovarian OR Ovary Cance OR Cancer, Ovary OR Cancers, Ovary OR Ovary Cancers OR Ovarian Cancer OR Cancer, Ovarian OR Cancers, Ovarian OR Ovarian Cancers OR Cancer of Ovary OR Cancer of the Ovary)  索引=SCI-EXPANDED, SSCI, A&HCI, CPCI-S, CPCI-SSH, BKCI-S, BKCI-SSH, ESCI, CCR-EXPANDED, IC 时间跨度=2015-2020 |
